# The fatty degeneration of the lumbar erector spinae muscles affects dynamic spinal compensation ability during gait in adult spinal deformity

**DOI:** 10.1038/s41598-021-97358-5

**Published:** 2021-09-10

**Authors:** Kousei Miura, Hideki Kadone, Tomoyuki Asada, Masao Koda, Toru Funayama, Hiroshi Takahashi, Hiroshi Noguchi, Kentaro Mataki, Yosuke Shibao, Kosuke Sato, Fumihiko Eto, Mamoru Kono, Kenji Suzuki, Masashi Yamazaki

**Affiliations:** 1 Department of Orthopaedic Surgery, Faculty of Medicine, University of Tsukuba, 1-1-1 Tennodai, Tsukuba, Ibaraki 305-8575 Japan; 2 Center for Innovative Medicine and Engineering, University of Tsukuba Hospital, 1-1-1 Tennodai, Tsukuba, Ibaraki 305-8575 Japan; 3 Center for Cybernics Research, University of Tsukuba, 1-1-1 Tennodai, Tsukuba, Ibaraki 305-8575 Japan

**Keywords:** Diseases, Medical research

## Abstract

This study aimed to investigate whether fat infiltration in lumbar paravertebral muscles assessed by magnetic resonance imaging (MRI) could be related to dynamic sagittal spino-pelvic balance during gait in adult spinal deformity (ASD). This is a retrospective analysis of 28 patients with ASD. The fat infiltration rate of lumbar erector spinae muscles, multifidus muscles and psoas major muscles was measured by T2 weighted axial MRI at L1-2 and L4-5. Dynamic sagittal spinal and pelvic angles during gait were evaluated using 3D motion analysis. The correlation between fat infiltration rate of those muscles with variations in dynamic kinematic variables while walking and static radiological parameters was analyzed. Spinal kyphosis and pelvic anteversion significantly increased during gait. Fat infiltration rate of erector spinae muscles at L1-2 was positively correlated with thoracic kyphosis (*r* = 0.392, *p* = 0.039) and pelvic tilt (*r* = 0.415, *p* = 0.028). Increase of spinal kyphosis during walking was positively correlated with fat infiltration rate of erector spinae muscles both at L1-2 (*r* = 0.394, *p* = 0.038) and L4-5 (*r* = 0.428, *p* = 0.023). Qualitative evaluation of lumbar erector spinae muscles assessed by fat infiltration rate has the potential to reflect dynamic spino-pelvic balance during gait.

## Introduction

Full-spine lateral standing radiographs are used widely to evaluate adult spinal deformities (ASD). To date, sagittal spino-pelvic parameters such as sagittal vertical axis (SVA), lumbar lordosis (LL), pelvic tilt (PT) and pelvic incidence (PI) are calculated to diagnose and consider surgical strategies for patients with ASD^1–3^. On the other hand, there has been increasing awareness of the dynamic changes of sagittal spino-pelvic alignment. That is because a full-spine lateral standing radiograph is only a static evaluation in a standing position. Moreover, several sagittal spino-pelvic parameters are affected by patient positioning^4^. In general, low back pain and gait difficulty in ASD patients tend to increase during continuous standing or walking. Questionnaires about health-related quality of life (HR-QOL) such as the Oswestry Disability Index (ODI) and short form 36-item health survey (SF-36) that are commonly used to evaluate clinical outcomes for ASD include many items related to dynamic motion.

To resolve issues related to static evaluations, three-dimensional (3D) gait analysis using motion capture systems for ASD patients have been used recently to evaluate dynamic spino-pelvic balance. A few clinical studies using gait motion analysis have reported that dynamic sagittal spino-pelvic balance deteriorated during walking in patients who have thoracolumbar spinal deformity^5,6^, indicating that dynamic evaluation of spinal sagittal balance may be an important addition to conventional full-spine lateral standing radiographs. However, it is difficult to perform 3D gait analysis as it requires special, and not always available, instrumentation.

Many approaches using various types of image examination have been proposed to evaluate the relationship between thoracolumbar kyphotic deformity and lumbar back muscles. Weakness of lumbar extensor muscles and marked atrophy of these muscles with fat infiltration can be demonstrated by isokinetic measurement and computed tomography (CT) scanning in patients with lumbar degenerative kyphosis^7^. A study using enhanced echo intensity on an ultrasound image of skeletal muscles found that increased intramuscular adipose tissue negatively correlated with muscle strength^8^. Patients with lumbar degenerative kyphosis have less muscularity of the lumbar extensor muscles and a higher proportion of fat deposits in these muscles compared to patients without spinal deformity, assessed using magnetic resonance imaging (MRI)^9^. Fat infiltration in back muscles has been considered a qualitative evaluation of the muscles. Although the relationship between static spinal alignment and back muscles has been reported recently in ASD patients, few studies have reported the relationship between dynamic sagittal spino-pelvic balance during gait and lumbar back muscles.

We hypothesized that the severity of fat infiltration in lumbar back muscles might reflect sagittal spino-pelvic imbalance during gait for patients with ASD. If so, the qualitative evaluation of lumbar back muscles might be an alternative option for assessing the dynamic spino-pelvic balance by three-dimensional gait motion analysis. The aim of this study was to investigate whether fat infiltration in lumbar paravertebal muscles assessed by MRI is related to dynamic sagittal spino-pelvic balance evaluated by three-dimensional gait motion analysis in ASD patients.

## Methods

### Subjects

A single institutional retrospective analysis was performed using MRI and 3D gait analysis data in ASD patients. ASD was diagnosed based on radiographic inclusion criteria measured on a full-spine lateral standing radiograph: PI-LL > 10°; SVA > 4 cm; and PT > 20° as parameters defining global sagittal malalignment according to the SRS-Schwab ASD Classification^1^. The inclusion criteria were ASD patients who fulfilled at least one of the classification criteria and underwent 3D gait analysis and lumbar MRI between 2015 and 2019. Patients who had problems walking caused by conditions such as severe lower limb arthritis, paresis or cardiopulmonary dysfunction were excluded from the present series. Approval from the ethics committee of our local ethics committee (Tsukuba Clinical Research and Development Organization) (approval number: H26-144) was obtained for this study design. The present study was performed in accordance with the contemporary amendments of the Declaration of Helsinki and within an appropriate ethical framework. Informed consent for both study participation and publication of identifying information/images in an online open-access publication was obtained from all patients.

### Radiological measurements

**Figure 1:**
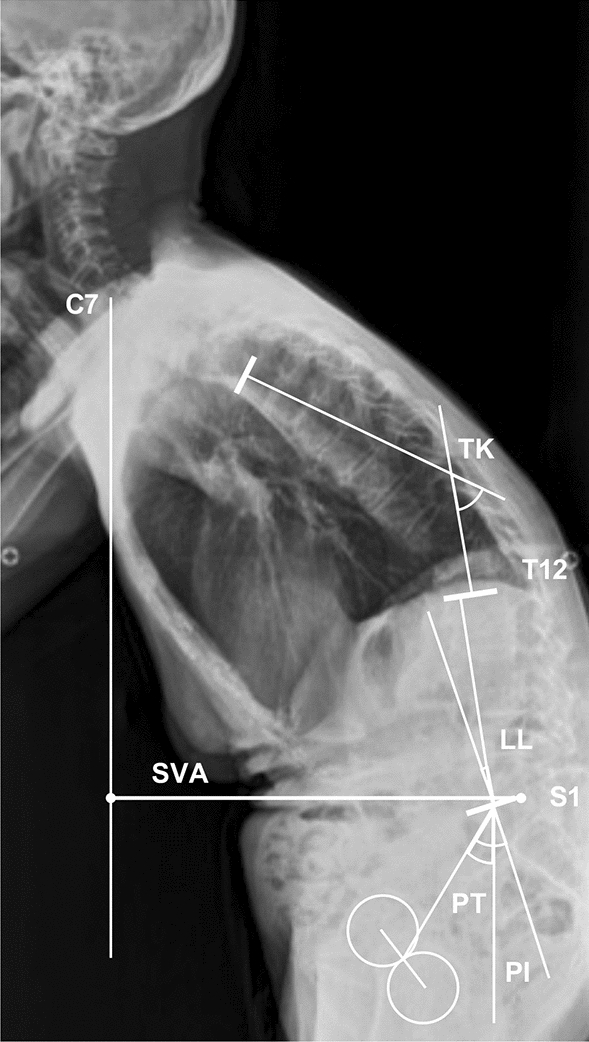
Static spino-pelvic parameters measured by full-spine lateral standing radiographs. SVA, the sagittal vertical axis; TK, thoracic kyphosis (T5-12); LL, lumbar lordosis (T12-S1); PT, pelvic tilt; PI, pelvic incidence.

Full-spine lateral standing radiographs were taken to evaluate static spino-pelvic alignment. Each patient was requested to place their fingertips on their clavicle and stand comfortably. The static spino-pelvic parameters thoracic kyphosis (TK), SVA, LL, PT, and PI-LL were measured (Fig. 1).

### Measurement of fat infiltration in lumbar paravertebral muscles on MRI

**Figure 2:**
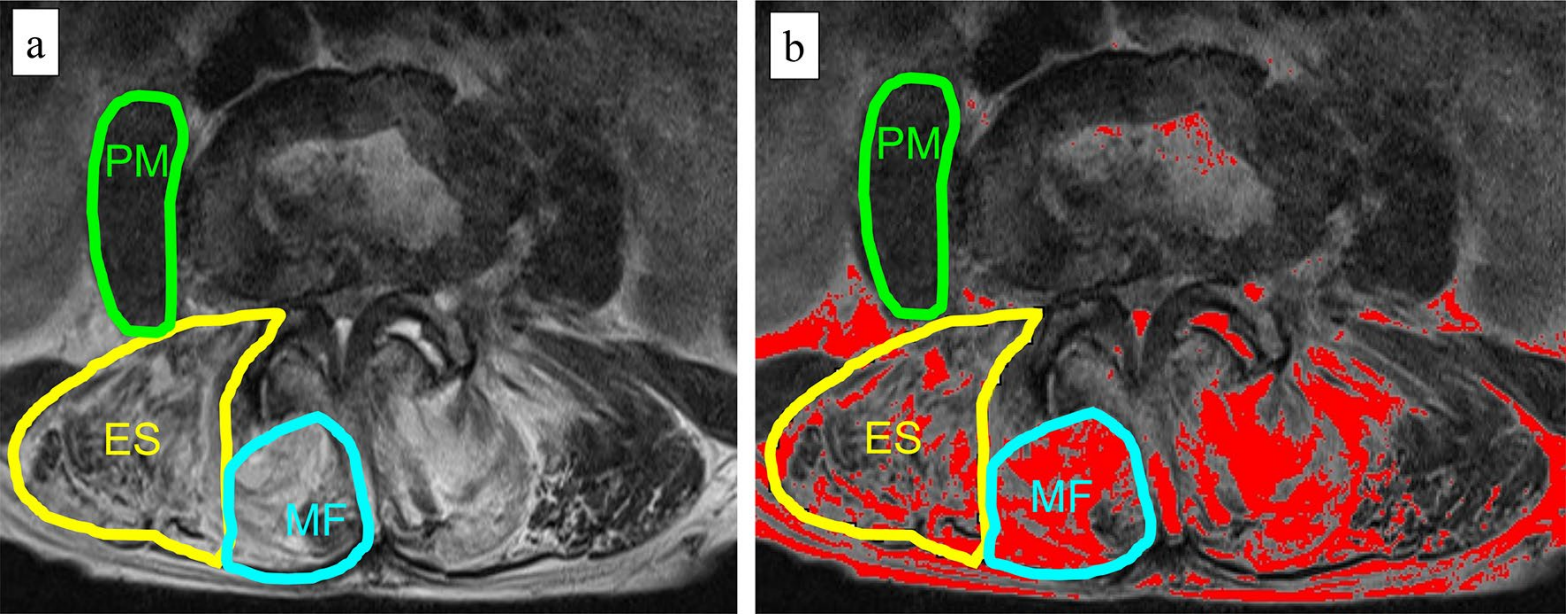
Calculation of the fat infiltration rate defined as the proportion of the area of the intramuscular fat tissue to the area of the regions of interest (ROI) of lumbar paravertebral muscles. (**a**) Manually traced regions of interest (ROI) around the erector spinae muscles, multifidus muscles and psoas major muscle at the L4-5 disc level on axial T2 weighted MRI images. (**b**) The intramuscular red area showing the fat signal area in the lumbar back muscles by using Image J software (National Institutes of Health, Bethesda, MD, USA). ES, erector spinae muscles; MF, multifidus muscles.

Muscle measurement data were collected with a 1.5T MRI scanner (Achieva, Philips Healthcare, Eindvohen, The Netherlands). T2 weighted axial MRI images were obtained at the L1-2 and L4-5 levels parallel to the intervertebral disc to measure fat infiltration of the lumbar paravertebral muscles. Image J (National Institutes of Health, Bethesda, MD, USA) software, which can distinguish fat tissue from muscle tissue by the differences of pixel brightness and calculates the number of pixels within assigned areas on the MRI image, was used for measurement. The regions of interest (ROI) around the bilateral erector spinae muscles and multifidus muscles at L1-2 and L4-5 levels were manually traced. The ROI around the psoas major muscles at L4-5 level were manually traced. A pixel grey-scale value > 120 on ImageJ was considered fat tissue in the lumbar paraspinal muscles^10^. The fat infiltration rate was defined as the proportion of the averaged right and left side areas of intramuscular fat tissue to the ROI of those muscles^11,12^ (Fig. 2). Two examiners independently evaluated the muscle measurement on MRI twice to assess the intra- and inter-observer reliability by using the intraclass correlation coefficients (ICC). Intra-observer reliability (ICC 0.924–0.975) and inter-observer reliability (ICC 0.937–0.990) showed excellent agreement.

### Three-dimensional gait analysis

**Figure 3:**
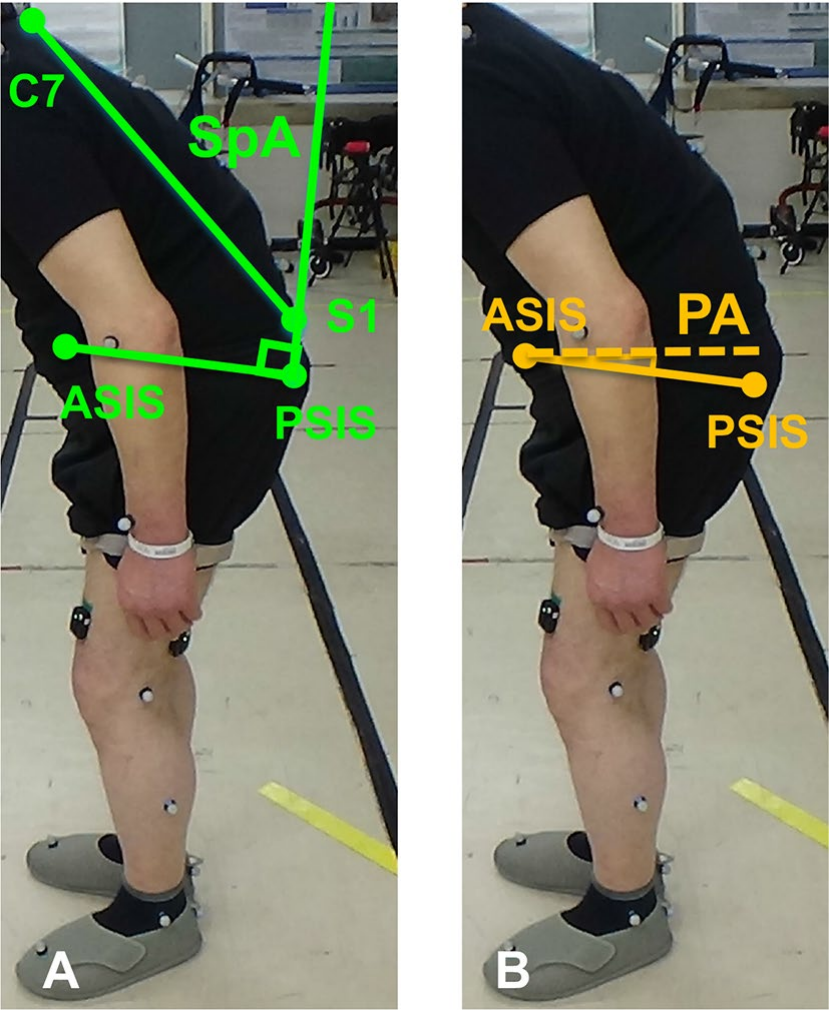
Measurement of dynamic spino-pelvic sagittal balance during gait from the three-dimensional motion analysis. (**A**) The sagittal spinal angle (SpA) is the sagittal angle between the line connecting the reflective markers on the C7 and S1 spinous process and the line connecting reflective markers on the anterior superior iliac spine (ASIS) and the posterior superior iliac spine (PSIS). (**B**) The sagittal pelvic angle (PA) is the sagittal angle between the horizontal axis and the line connecting the reflective markers on the ASIS and PSIS).

A laboratory with a 25 m-long oval-shaped flat walkway was available in our hospital for gait analysis. Gait motion was analyzed using a Vicon MX system (Vicon, Oxford, UK). The VICON 16-camera system tracked movement of reflective markers attached to the spinous processes and pelvis of the patients. The patients were instructed to walk at a comfortable pace for as long as possible on the oval-shaped walkway until they stopped walking because of fatigue. We evaluated dynamic spinal and pelvic sagittal angles from this motion capture system. Dynamic spino-pelvic parameters were defined as follows: the sagittal spinal angle (SpA, the sagittal angle between the line connecting the reflective markers on the C7 and S1 spinous process and the line connecting reflective markers on the anterior superior iliac spine (ASIS) and the posterior superior iliac spine (PSIS)); and the sagittal pelvic angle (PA, the sagittal angle between the horizontal axis and the line connecting the reflective markers on the ASIS and PSIS) (Fig. 3). As for PA, pelvic anteversion was defined as plus (+), and pelvic retroversion was defined as minus (−).

### Statistical analysis

Wilcoxon signed-ranks tests were used to compare dynamic spino-pelvic kinematic variables (SpA and PA) between the first lap and the final lap on the walkway. Spearman correlation coefficients were used to analyze the relationship between the fat infiltration rate of the three lumbar paravertebral muscles with the variations in dynamic spino-pelvic kinematic variables while walking (ΔSpA and ΔPA), and with the static radiological spino-pelvic parameters (TK, LL, PT and PI-LL). All statistical analyses were performed using JMP (version 14.0.0; SAS Institute Inc, Cary, NC, USA). *p* values < 0.05 were considered significant.

## Results

### Patient demographics

A total of 28 patients (8 male and 20 female) with ASD were enrolled in the present study. The mean age of the patients was 69 ± 7.6 years old (range 55–84 years old) and mean the BMI was 23.3 ± 3.0 (range 17.1–29.7). The radiological diagnoses were degenerative lumbar kyphosis in 10 cases, flatback syndrome in 8 cases, and degenerative lumbar kyphoscoliosis in 10 cases.

### Radiological evaluation

The mean values for static radiological spino-pelvic parameters measured from full-spine lateral standing radiographs were: SVA, 108 ± 52 mm (range − 11–238 mm); TK, 23° ± 16° (range − 4.4°–57°); LL, 12° ± 18° (range − 21°–49°); PT, 33° ± 13° (range 7°–63°); PI, 48° ± 12° (range 18°–71°); PI-LL, 36° ± 17° (range 1°–75°).

### Evaluation of fat infiltration rate in lumbar paravertebral muscles

The mean fat infiltration rate of erector spinae muscles at the L1-2 and L4-5 levels were 11.4 ± 11.8% and 12.9 ± 9.7%, respectively. The mean fat infiltration rate of multifidus muscles at these levels were 18.5 ± 15.2% and 24.1 ± 16.8%, respectively. The mean fat infiltration rate of psoas major muscles at L4-5 level were 1.8 ± 2.9%. The fat infiltration rate of multifidus muscles was significantly greater than that of the erector spinae muscles at both levels (*p* < 0.01).

### Dynamic spino-pelvic balance evaluation

The mean SpA was 17.8° ± 11.8° after the first lap and 19.5° ± 12.1° after the final lap (*p* < 0.01). The mean PA was 2.93° ± 7.94° after the first lap, and 4.39° ± 8.38° after the final lap (*p* < 0.01). Spinal kyphosis and pelvic anteversion significantly increased after patients walked to fatigue.

### Correlation analysis

The fat infiltration rate of erector spinae muscles at L1-2 was significantly correlated with TK (*r* = 0.392, *p* = 0.039) and PT (*r* = 0.415, *p* = 0.028) (Table 1). In contrast, there was no correlation between the fat infiltration rate of multifidus muscles and static spino-pelvic parameters. The increase of spinal kyphosis during walking was positively correlated with the fat infiltration rate of the erector spinae muscles both at L1-2 (*r* = 0.394, *p* = 0.038) and at L4-5 (*r* = 0.428, *p* = 0.023) (Table 2). No correlation was found between the fat infiltration rate of other muscles and dynamic spino-pelvic balance.

**Table 1:** Relationship between the fat infiltration rate in lumbar paravertebral muscles and static radiological spino-pelvic parameters.

		SVA	TK	LL	PT	PI-LL
**Erector spinae muscles**						
L1-2	*r*	0.026	**0.393**	0.033	**0.415**	0.135
	*p*-value	0.895	0.039*	0.867	0.028*	0.493
L4-5	*r*	0.051	0.292	− 0.019	0.156	0.031
	*p*-value	0.798	0.131	0.924	0.429	0.877
**Multifidus muscles**						
L1-2	*r*	− 0.001	0.315	0.020	0.209	0.027
	*p*-value	0.995	0.103	0.920	0.285	0.892
L4-5	*r*	− 0.014	0.367	0.196	0.339	0.070
	*p*-value	0.944	0.050	0.318	0.077	0.724
**Psoas major muscles**						
L4-5	*r*	− 0.257	− 0.158	0.267	− 0.278	− 0.265
	*p*-value	0.186	0.422	0.169	0.152	0.174

*SVA* sagittal vertical axis, *TK* thoracic kyphosis, *LL* lumbar lordosis, *PT* pelvic tilt, *PI-LL* pelvic incidence minus lumbar lordosis, *r* correlation coefficient.

*p < 0.05. Significant correlation shown in bold.

**Table 2:** Relationship between the fat infiltration rate in lumbar paravertebral muscles and the variations in dynamic spino-pelvic kinematic variables.

		ΔSpA	ΔPA
**Erector spinae muscles**			
L1-2	*r*	**0.394**	0.330
	*p*-value	0.038*	0.086
L4-5	*r*	**0.428**	0.239
	*p*-value	0.023*	0.222
**Multifidus muscles**			
L1-2	*r*	0.220	0.292
	*p*-value	0.260	0.132
L4-5	*r*	0.314	0.100
	*p*-value	0.104	0.613
**Psoas major muscles**			
L4-5	*r*	− 0.121	0.534
	*p*-value	− 0.135	0.493

*SpA* sagittal spinal angle, *PA* sagittal pelvic angle, *r* correlation coefficient.

*p < 0.05. Significant correlation shown in bold.

## Discussion

**Figure 4:**
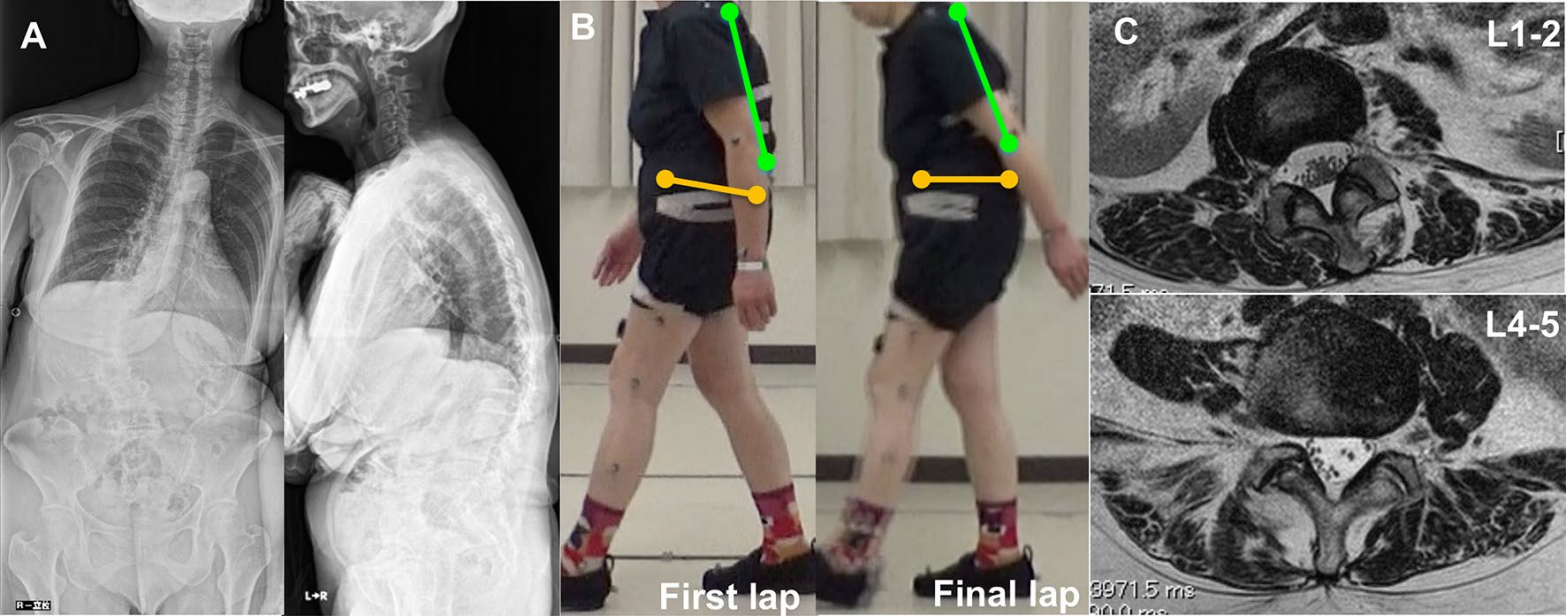
A representative case of a 63-year-old female patient with almost normal TK and increased PT, large fat infiltration rate of the lumbar erector spinae muscles and an increase of spinal kyphosis and pelvic anteversion. (**A**) Full-spine radiographs. SVA: 89 mm, TK: 40°, LL5.8°, PT 35°, PI: 39° (**B**) Posture during the three-dimensional gait motion analysis on the first and final laps. ΔSpA: 2.5°, ΔPA: 3.1° (**C**) T2 weighted axial MRI images at L1-2 and L4-5. The fat infiltration rate of the lumbar erector spinae muscles (L1-2: 15% and L4-5: 24%).

In general, decrease of thoracic kyphosis and increase of pelvic retroversion work as compensatory mechanisms for decreased lumbar lordosis in ASD patients^13^. Static sagittal spino-pelvic parameters measured by full-spine lateral standing radiographs are influenced by transient compensations for spinal deformity and may underestimate spinal balance^5^. As previously reported, 3D gait analysis demonstrated that spinal kyphosis and pelvic anteversion significantly increased while walking and detected a failure of compensation for ASD^6^. Although 3D gait analysis could be optimal for quantitatively evaluating spinal balance, it has a high initial cost, making its broad use difficult. Thus, another method of static evaluation that can predict the dynamic change of spinal balance is needed. We focused on analyzing fatty degeneration of the lumbar paravertebral muscles using MRI, which is both convenient and static. As a result, the current analysis revealed significant positive correlation between increased spinal kyphosis during gait and fat infiltration in the lumbar erector spinae muscles (Fig. 4).

There has been increasing recognition of the benefits of evaluating the back muscles in patients with spinal disorders such as spinal deformity. Recent studies have mainly reported the relationship between fatty degeneration of the back muscles and static radiological spino-pelvic parameters. Fat infiltration in the lumbar back muscles has been correlated with several static spino-pelvic parameters including SVA^14^, PT^15^ and LL^16^. Regarding the difference of fatty degeneration in lumbar back muscles between lumbar levels, fatty degeneration in lumbar back muscles at L4-5 was more severe than L1-2 in this study. Similarly, previous studies have shown that fatty degeneration at the lower lumbar levels was worse than at the upper lumbar levels^12,17^. That is because lower lumbar levels are closer to the fixed pelvis. Thus, the lower lumbar levels may be subjected to higher mechanical stress than lower lumbar levels^17^. Furthermore, it also has been reported that fatty degeneration of lumbar back muscles is correlated with postoperative outcomes for ASD. The increase of fat infiltration in back muscles assessed by preoperative MRI may predict proximal junctional kyphosis^18^ and nonunion of the lumbosacral junction^19^. In addition, a very limited number of studies have reported on the correlation between fatty degeneration of the back muscles and the dynamic spinal balance while walking. Lee et al. reported that dynamic spinal balance parameters assessed using 3D gait analysis showed greater correlation with fat infiltration of the lumbar paraspinal extensor muscles than with static radiological parameters^20^. It seems that the correlation between fatty degeneration of the back muscles and the dynamic spinal balance while walking might be important because symptoms such as back pain and gait difficulty in ASD patients increase while walking.

In the present study, the increase of spinal kyphosis during walking was positively correlated with the fat infiltration rate of the lumbar erector spinae muscles at both L1-2 and L4-5. The increased fatty degeneration in lumbar erector spinae muscles could cause severe failure of spinal compensation during walking in ASD. Thus, evaluation of the fat infiltration of lumbar erector spinae muscles has the potential to reflect the dynamic change in spinal sagittal balance in ASD patients. In addition to conventional static radiological examination, it might be useful to detect individual differences in dynamic spinal compensation by analyzing the fatty condition of the lumbar erector spinae muscles to better understand the individual pathophysiology of each ASD patient. Although three-dimensional gait motion analysis is ideal for evaluating the difference of sagittal spino-pelvic balance during gait between individuals, it is hard to commonly evaluate it because of the high initial cost. On the other hand, MRI is commonly used in ASD patients. Therefore, it can be a useful alternative option for detecting individual differences in sagittal spino-pelvic balance by analyzing the fatty condition of the lumbar erector spinae muscles on MRI.

There are several limitations to this study. First, the study is limited by the small sample size, which cannot be generalized for all patients with ASD. It has been recently reported that fat infiltration in the lumbar back muscles was correlated with static spino-pelvic parameters including SVA^14^ and LL^16^ unlike in this study. To confirm that, further study including larger sample size is needed. Second, degenerative lumbar scoliosis was not excluded in this study. There was a possible effect of the scoliotic curve on paraspinal muscle quality. However, a previous study has reported that the scoliotic curve affected not the laterality of fatty infiltration of bilateral paravertebral muscles but the laterality of the cross-sectional area (CSA)^11^. Thus, we considered evaluating the fat infiltration of paravertebral muscles was a more accurate method than evaluating the CSA in patients with ASD, including degenerative lumbar scoliosis. Third, we evaluated fat infiltration only of the lumbar paravertebral muscles. It has been reported that fatty degeneration of hip and knee joint muscles is correlated with static radiological spino-pelvic parameters^21^. Hence, fat infiltration of hip and knee joint muscles may be correlated with dynamic spino-pelvic balance. Furthermore, back muscle strength and sarcopenia, which may affect the evaluation of back muscles as well, were not assessed in this study. A prospective study with a larger sample using these variables should be completed in the future to elucidate the role of those muscles for dynamic spino-pelvic balance.

## Conclusions

In this study, the fat infiltration rate of lumbar back muscles was compared with static spino-pelvic alignment parameters and dynamic spino-pelvic balance. The fat infiltration rate of the lumbar erector spinae muscles both at L1-2 and L4-5 was positively correlated with the increase of spinal kyphosis during walking assessed using 3D gait analysis, indicating that more severe failure of spinal compensation during walking could occur in ASD patients with greater fatty degeneration of the lumbar erector spinae muscles. These findings suggest that the qualitative evaluation of lumbar erector spinae muscles assessed by fat infiltration rate has the potential to reflect the ability for spinal compensation in individual ASD patients. In addition to conventional static radiological examination, it might be useful to detect individual differences in spinal compensation by analyzing the fatty condition of the lumbar erector spinae muscles.
